# Outcome comparison of radical prostatectomy versus seed brachytherapy for clinically localized prostate cancer using two biochemical recurrence definitions

**DOI:** 10.1186/s12893-023-02121-4

**Published:** 2023-08-27

**Authors:** Xue-hua Zhu, Fan Zhang, Ze-nan Liu, Ji-de He, Zi-ang Li, Lu-lin Ma, Yi Huang, Jian Lu

**Affiliations:** 1 Department of Urology, Peking University Third Hospital, Beijing, China; 2 Department of Urology, Shandong Cancer Hospital and Institude, Shandong First Medical University and Shandong Academy of Medical Sciences, Jinan, China

**Keywords:** Localized prostate cancer, Radical prostatectomy, Seed brachytherapy, Biochemical recurrence definition

## Abstract

**Objective:**

We compared the outcome of radical prostatectomy (RP) with seed brachytherapy (BT) in clinically localized prostate cancer (LPCa) using two different biochemical recurrence (BCR) definitions.

**Methods:**

Clinical data of 1117 patients with non-metastatic prostate cancer (PCa) treated with either RP or BT as the basis of the multimodal therapy from a single tertiary hospital between 2007 and 2021 were retrospectively analyzed. 843 LPCa patients (RP = 737, BT = 106) with at least one prostate-specific antigen (PSA) test after treatment were finally included. The BCR survival was evaluated by direct comparison and one-to-one propensity score matching (PSM) analysis using surgical definition (PSA ≥ 0.2ng/ml) for RP and surgical/Phoenix definition (PSA nadir + 2ng/ml ) for BT. The propensity score (PS) was calculated by multivariable logistic regression based on the clinicopathological parameters.

**Results:**

Median follow-up was 43 months for RP patients and 45 months for BT patients. Kaplan–Meier analysis did not show any statistically significant differences in terms of BCR-free survival (BFS) between the two groups when using Phoenix definition for BT (*P* > 0.05). Similar results were obtained in all D’Amico risk groups when stratified analyses were conducted. However, RP achieved improved BFS compared to BT in the whole cohort and all risk groups with the surgical definition for BT(*P* < 0.05). After adjusting PS, 192 patients were divided into RP and BT groups (96 each). RP presented a better BFS than BT when using the surgical definition (*P* < 0.001), but no significant difference was found when using the Phoenix definition (*P* = 0.609).

**Conclusion:**

Inconsistent BCR-free survival outcomes were acquired using two different BCR definitions for BT patients. RP provided comparable BFS with BT using the Phoenix definition but better BFS using the surgical definition, regardless of whether the PSM was performed. Our findings indicated that an exact BCR definition was critical for prognostic assessment. The corresponding results will assist physicians in pretreatment consultation and treatment selection.

## Background

Prostate cancer (PCa) is a major health concern currently ranked first among newly diagnosed malignant tumors in Western countries [1]. Due to the widespread use of prostate-specific antigen (PSA) screening, the incidence rate of PCa is gradually increasing in China [2]. Radical prostatectomy (RP) and seed brachytherapy (BT) are two primary therapeutic modalities for clinically localized prostate cancer (LPCa). Adjuvant androgen deprivation therapy (ADT) and radiotherapy (RT) are beneficial complementary options for individuals receiving RP or BT as a definitive treatment strategy [3]. However, prospective randomized controlled trials are still lacking to compare the efficacy of RP- and BT-based treatment approaches for LPCa in the multimodal era [4]. Retrospective studies and systematic reviews yield inconclusive results, making it difficult for patient consultation and treatment selection [5, 6].

Biochemical recurrence (BCR) may be a precursor to local and distant recurrence after curative treatment, which leads to shorter cancer-specific survival (CSS) [7]. BCR is defined differently depending on the treatment modality. The American Urologic Association (AUA) defines BCR after RP as a total PSA (tPSA) > 0.2 ng/mL, and the American Society for Therapeutic Radiology and Oncology (ASTRO) and Radiation Therapy Oncology Group define BCR after RT as the nadir tPSA + 2 ng/ml, which is known as the Phoenix criteria [8, 9]. However, BT can generate high doses of radiation that can produce effects comparable to prostate removal. Therefore, a series of studies attempted to assess the efficacy of BT with the surgical BCR standard [10, 11].

In this study, we compared the outcomes of RP and BT patients from a single institution with two BCR criteria due to the nonuniform BCR definition for BT patients. One-to-one propensity score matching (PSM) was utilized to minimize the baseline difference to equalize the characteristics of RP and BT patients.

## Methods

### Patients selection

A total of 1117 patients with non-metastatic PCa treated with RP or BT with or without androgen deprivation therapy (ADT) and external beam radiotherapy (EBRT) from Peking University Third Hospital between 2007 and 2021 were retrospectively analyzed. Two hundred and seventy-four patients were excluded because of complete information absence(n = 6), loss of follow-up (n = 154), Neoadjuvant androgen deprivation therapy (Neo-ADT, n = 70), T4 (n = 6), PSA persistence(n = 33), and drug trial (n = 5). PSA persistence is defined as no tPSA value below the BCR standard six weeks after the treatment. Finally, eight hundred and forty-three LPCa patients (RP = 737, BT = 106) with at least one PSA test after the treatment were included in our study.

### BCR definition and analysis process

PSA test was performed monthly within three months after initial treatment. The follow-up plan depends on the PSA results and the urologists’ experience. In general, it is recommended that patients be followed up every three to six months for the first year and once every six months thereafter. Surgical BCR is defined as tPSA > 0.2 ng/mL after curative treatment, and a rise of 2 ng/mL or more above the nadir tPSA after BT with or without ADT is regarded as Phoenix BCR. The scenario of continuous PSA increase triggering salvage treatment is also considered BCR in both RP and BT patients.

**Fig. 1:**
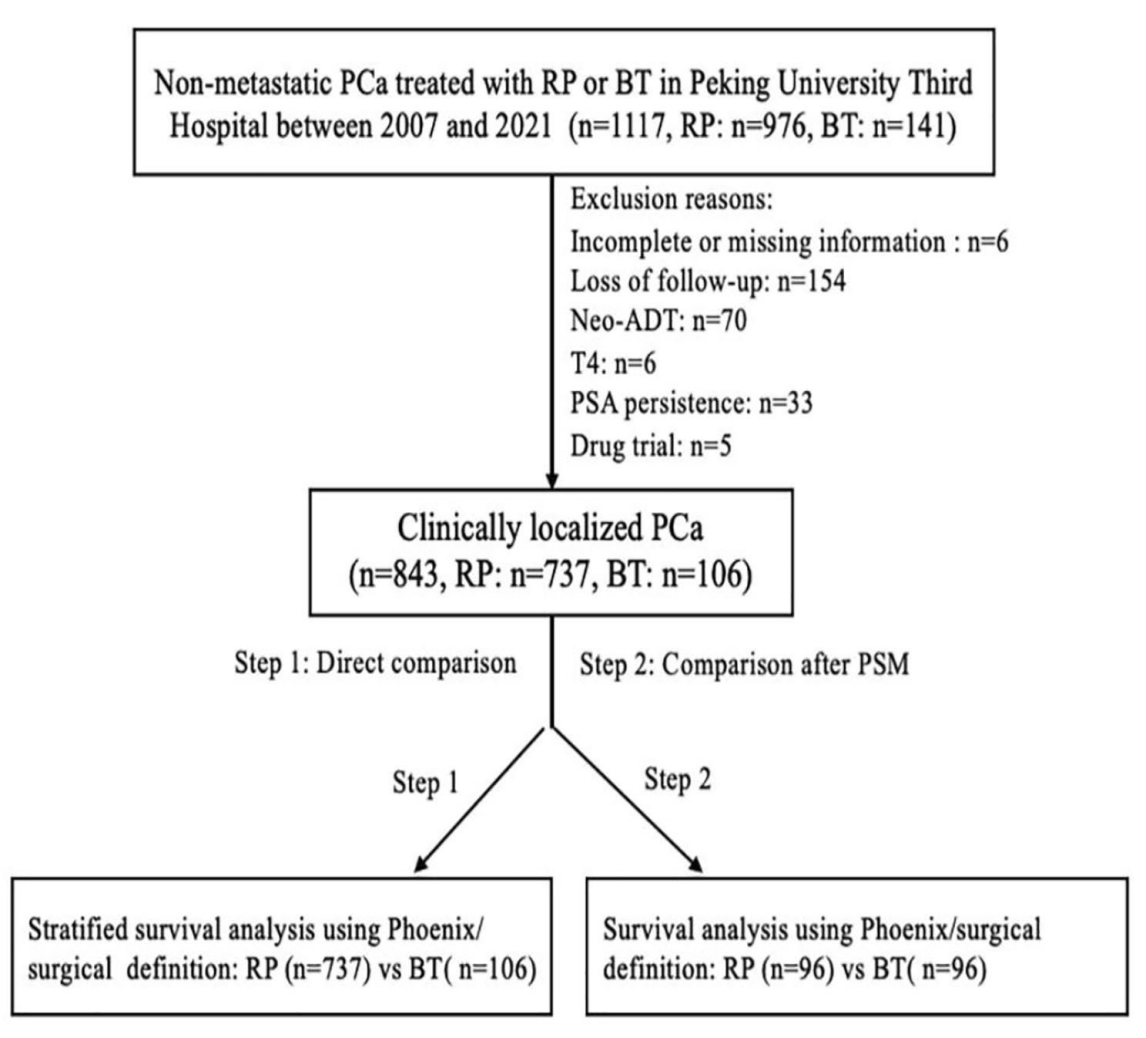
Patients selection and analysis process

We first evaluated the BCR-free survival (BFS) across the two therapeutic interventions using the surgical criteria for RP and the Phoenix definition for BT. Then, we compared the BFS between the two groups utilizing the surgical standard for both RP and BT. A stratified analysis was performed according to the D’Amico risk criteria. One-to-one PSM analysis was further conducted to balance the baseline characteristics, including age (continuous data), tPSA (continuous data), Gleason score (GS, ranked data), clinical T stage (cT-stage, ranked data), percentage of positive biopsy (PPB, continuous data), and Charlson score (ranked data). The patient selection and analysis process is shown in Fig. 1.

### Treatment protocol for RP and BT

All RP procedures were performed by minimally invasive laparoscopy. Standard or extended lymph node dissection was performed in intermediate and high-risk patients based on the D’Amico criteria. EBRT ± ADT was prescribed to patients with adverse pathology (pT3, positive surgical margin).

I-125 was used for all patients. The three-dimensional (3D) treatment planning system (TPS) and quality verification system for BT are manufactured by Prowess 3D Version 3.02 3D TPS machine produced by SSGI Company of the United States. TPS was utilized to determine the number of seeds and the radiation dose based on the prescribed dose of 145 Gy. Cross-sectional images of the prostate from the base to the apex were acquired by intraoperative transrectal ultrasound. Imaging information was then transmitted to the TPS to reconstruct the 3D shape of the prostate. The implant needle was guided to the correct position with the assistance of the guidance system. The seeds were individually deposited using a Mick Applicator®. BT permanently implants radioactive sources, which generate radiation continuously with therapeutic effects for six months. Patients with intermediate- and high-risk PCa were recommended ADT with a duration of 3–6 months and 2–3 years, respectively. BT patients with unsatisfied PSA decline were considered for EBRT.

### Statistical analysis

Continuous variables with abnormal distribution were presented as median (quartile), and categorical and ranked data were shown as numbers (percentage). The Mann–Whitney U test was performed to determine statistical significance for ranked variables and continuous variables with abnormal distribution. The propensity score (PS) was calculated using multivariable logistic regression based on age, tPSA, GS, PPB, cT stage, and Charlson score. All statistical analyses were performed using SPSS version 27.0. Two-sided *P* < 0.05 was considered statistically significant. The Kaplan–Meier and log-rank statistics were used to estimate the BFS.

## Results

The median follow-up was 43 months for RP patients and 45 months for BT patients. The baseline characteristics are listed in Table 1. Eight hundred and forty-three LPCa patients treated by either RP (n = 737) or BT(n = 106) with a median follow-up of 45 months (range: 1-170) after treatment were included in our study. There were significant differences between the two groups in age, clinical T stage (cT stage), Charlson score, and D’Amico risk (all *P* < 0.05).

**Table 1:** Characteristics of RP and BT patients

	RP (n = 737)	BT (n = 106)	*P* value
Age(years)	69.0 (64.0–75.0)	78.00 (72.0–81.0)	< 0.001
tPSA(ng/ml)	11.28 (7.32–19.03)	12.13 (7.09–25.89)	0.173
Gleason score(%)			0.426
6	174 (23.6%)	22 (20.8%)	
7	266 (36.1%)	34 (32.1%)	
8	146 (19.8%)	31 (29.2%)	
9	131 (17.8%)	16 (15.1%)	
10	20 (2.7%)	3 (2.8%)	
PPB	0.42 (0.25–0.58)	0.44 (0.25–0.67)	0.089
Clinical T stage (%)			0.003
T1	28 (3.8%)	5 (4.7%)	
T2	461 (62.6%)	81 (76.4%)	
T3	248 (33.6%)	20 (18.9%)	
Charlson score (%)			0.001
0	542 (73.5%)	64 (60.4%)	
1	162 (22.0%)	27 (25.5%)	
2	24 (3.3%)	13 (12.3%)	
3	8 (1.1%)	1 (0.9%)	
4	1 (0.1%)	1 (0.9%)	
D’Amico risk (%)			< 0.001
Low	26 (3.5%)	13 (12.3%)	
Intermediate	118 (16.0%)	29 (27.4%)	
High	593 (80.5%)	64 (60.4%)	

PPB: percentage of positive biopsy

**Fig. 2:**
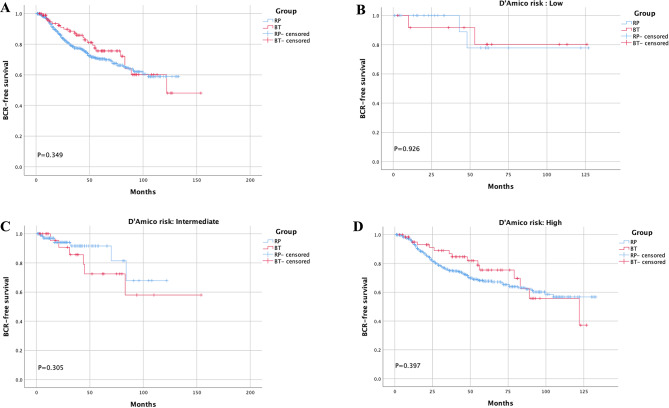
Direct comparison between RP and BT using Phoenix definition for BT in whole cohort **(A).** Stratified analysis according to D’Amico risk criteria using Phoenix definition **(B-D)**.

**Fig. 3:**
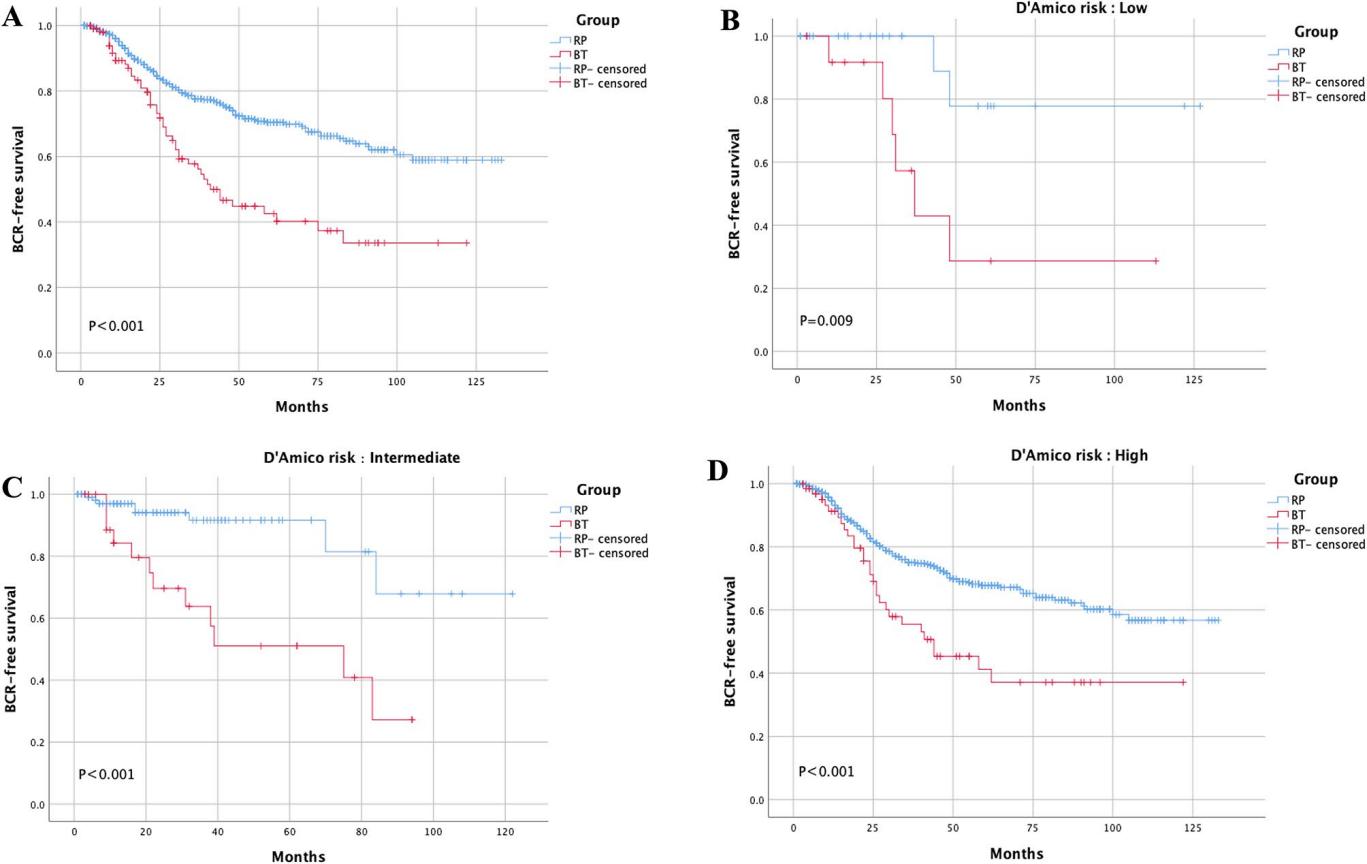
Direct comparison using surgical definition for RP and BT in whole cohort **(A).** Stratified analysis according to D’Amico risk criteria using surgical definition **(B-D)**.

Inconsistent outcomes were acquired when using two different BCR definitions for BT patients. When the BFS rate was calculated using the Phoenix definition for BT, no significant difference was found between the two groups (Fig. 2A). Stratified analysis based on D’Amico risk acquired similar results(Fig. 2B-D). The 3- and 5-year BFS rates were comparable with the Phoenix definition for BT in the two groups (3-year BFS rate: RP vs. BT = 87.4% vs. 75.6%, 5-year BFS rate: RP vs. BT = 77.6% vs. 70.4%, both *P* > 0.05). When the BFS was compared with the surgical definition for both RP and BT, RP presented a better BFS than BT in the whole cohort (Fig. 3A) and all risk groups (Fig. 3B-D). The 3-and 5-year BFS rates of RP and BT were 77.6% vs. 57.7% (*P* < 0.001) and 70.4% vs. 42.6% (*P* < 0.001), respectively.

**Fig. 4:**
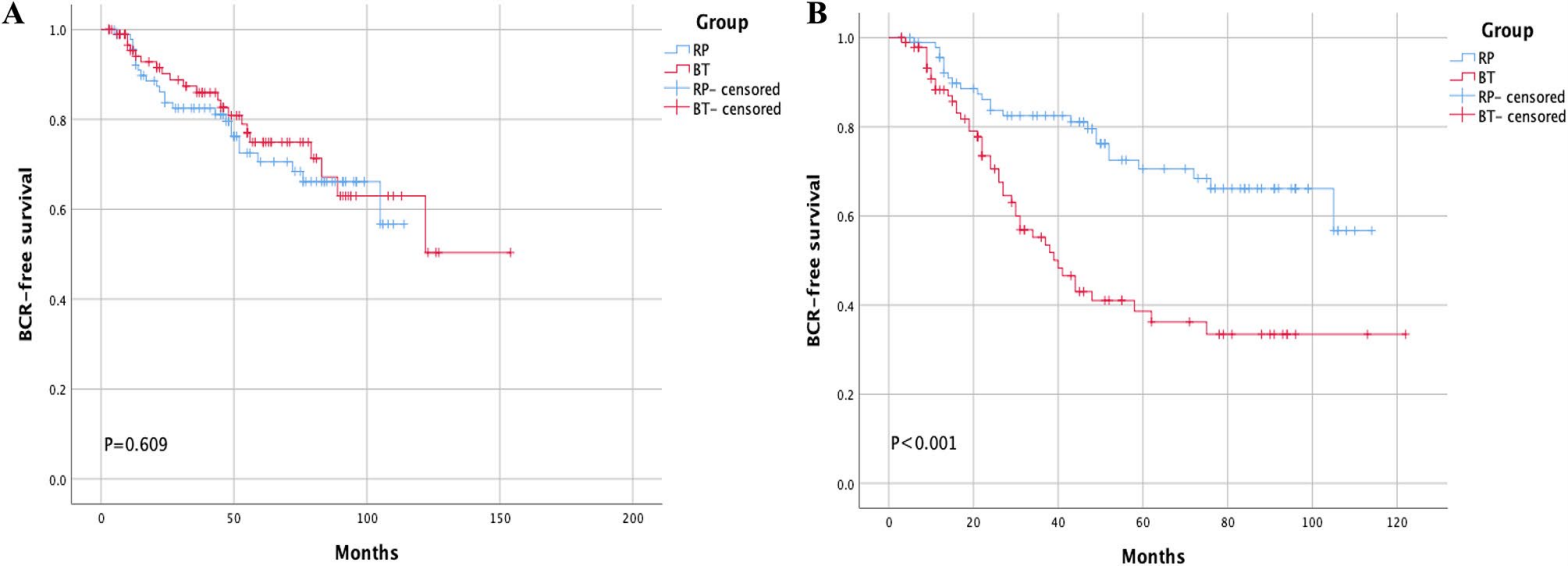
Comparison between RP and BT using the Phoenix definition for BT after PSM **(A).** Comparison between RP and BT using the surgical definition after PSM **(B)**

Ninety-six pairs were selected by PSM with a 1:1 ratio. The characteristics are presented in Table 2. The Kaplan-Meier showed a statistically significant difference regarding BFS rate in the two groups with the surgical definition (Fig. 4 B. 3-year BFS rate: RP vs. BT = 82.4% vs. 51.5%, 5-year BFS rate: RP vs. BT = 59.8% vs. 29.9%, both *P* < 0.001) but not with the Phoenix definition (Fig. 4A. 3-year BFS rate: RP vs. BT = 82.4% vs. 85.9%, 5-year BFS rate: RP vs. BT = 59.8% vs. 59.1%, both *P* > 0.05 ).

**Table 2:** Characteristics of RP and BT patients after PSM

	RP (n = 96)	BT (n = 96)	*P* value
Age(years)	77.00 (74.00–80.00)	77.00 (72.00–80.00)	0.988
tPSA(ng/ml)	10.68 (6.95–21.42)	11.91 (6.93–25.58)	0.399
Gleason score(%)			0.668
6	29 (30.2%)	20 (20.8%)	
7	22 (22.9%)	32 (33.3%)	
8	25 (26.0%)	27 (28.1%)	
9	19 (19.8%)	14 (14.6%)	
10	1 (1.0%)	3 (3.1%)	
PPB	0.40 (0.22–0.61)	0.44 (0.24–0.67)	0.117
Clinical T stage (%)			0.637
T1	4 (4.2%)	4 (4.2%)	
T2	70 (72.9%)	73 (76.0%)	
T3	22 (2.9%)	19 (19.8%)	
Charlson score (%)			0.601
0	65 (67.7%)	62 (64.6%)	
1	24 (25.0%)	25 (26.0%)	
2	6 (6.3%)	7 (7.3%)	
3	1 (1.0%)	1 (1.0%)	
4	0	1 (1.0%)	

## Discussion

RP and BT are two crucial definitive strategies for LPCa patients. With BCR as the endpoint in this study, we compared the outcomes of the two therapeutic interventions. Due to the lack of a consensual or widely accepted standard for BCR definition after BT, our research adopted two definitions, including the Phoenix and surgical standards [11, 12]. Different criteria resulted in different prognostic findings of BT, which influenced the outcome comparison with RP. The BFS did not differ significantly across the two modalities when utilizing the Phoenix criteria, even after adjusting the PS to balance the baseline parameters. When surgical criterion was utilized, however, the BFS of RP was better than that of BT through all risk groups. The same outcome was found after PSM.

Although it is still debatable whether surgical definition can be considered for BCR evaluation after BT, several studies have been carried out to assess the prognosis of BT patients using surgical criteria. Compared to nadir + 2, the PSA > 0.2 standard is a stricter and more sensitive criterion resulting in a considerable decrease in BFS as demonstrated by prior findings [13]. Tanaka et al. evaluated the BFS rate based on a cohort of 203 patients with organ-confined PCa. The 5-year BFS rate in patients with the Phoenix definition was 92.8%, and the surgical definition was 74.1% [10]. Gul et al. concluded that there were significant differences between the Phoenix and surgical BFS rates at five years and ten years but not at 15 years after BT therapy. This reminded us that a long enough time was necessary to fully assess the prognosis in BT patients [14]. Similar results were observed in our cohort. Compared to the Phoenix standard, the 3-year and 5-year BFS rates for BT patients based on the surgical standard were dramatically lower than the Phoenix standard-based BFS rates.

Prognostic comparison following RP- and BT-based treatment is not yet supported by high-quality evidence. Retrospective studies and meta-analysis currently demonstrated conflicting results about overall survival (OS), CSS, and BFS [15–18]. The inconclusive results may be due to the influence of adjuvant therapy, risk stratification, and different definitions of BCR [19–21]. Tsumura et al. investigated the prognosis of 214 pairs of intermediate-risk PCa patients treated with RP alone versus BT ± EBRT using PSM analysis. The results revealed an 8-year BFS benefit for BT when using the Phoenix criteria (87.4%vs. 74.3%, HR: 0.420, 95% CI: 0.273–0.647), while no significant difference was detected when using the surgical definition (76.7%vs. 74.3%, HR: 0.913, 95% CI: 0.621–1.341) [3]. The comparative analysis performed by Grimm et al. reported similar results, demonstrating that BT using the Phoenix definition delivered a better BFS than RP in PCa patients with low- and intermediate-risk [22]. Hayashi et al. conducted a retrospective analysis involving 588 LPCa patients following RP and BT (299 each) after PSM. It was determined that BT ± EBRT ± ADT produced comparable results to RP using the Phoenix definition in terms of overall survival (*P* = 0.429) but improved BFS (*P* = 0.003) in the intermediate-risk group [23]. Goy et al. proposed that patients with intermediate-risk PCa undergoing BT had a better BFS than RP, and a similar result was acquired after subset analysis in unfavorable PCa. The adjusted 10-year BFS was 80.2% for BT and 57.1% for RP in the study [24]. Our findings differ from those of the studies mentioned above. The present study showed that the BFS of RP was superior to BT when a lower PSA failure threshold was used for BT. However, there was no significant difference in BFS between RP and BT when using a higher threshold for BT in all risk groups.

In this study, surgery standard led to a decreased BFS in the BT group. The primary reason for this is that the surgery standard appears to be stricter for BT patients. Even though BT can kill tumor cells, it fails to achieve a low PSA level comparable to radical prostatectomy. Despite its widespread use in clinical practice and research, the Phoenix standard was originally set for EBRT. In addition, previous studies have supported the use of surgery criteria for patients with BT [10, 11]. To conduct a more thorough and objective comparison, our study utilized two BCR criteria. The study’s finding reminds urologists to clarify the BCR criteria when comparing the prognosis of RP and BT in clinical practice and patient prognosis consultations. In addition, the BCR standards relate to the timing of adjuvant therapy initiation. In general, the surgical standard implies initiating adjuvant treatment earlier. It is essential to clarify the criteria for BCR during the follow-up process to determine the optimal timing of adjuvant therapy.

There are some limitations to our study. Firstly, this is a retrospective with a proportion of loss of follow-up and selection bias. Secondly, RP is the preferred therapy option for non-elderly PCa patients without severe comorbidities in our institution, resulting in a relatively limited number of patients undergoing BT. Nonetheless, the findings of this study appear to favor this management route, as the BFS of RP is superior to that of BT when a stricter BCR criterion was applied. In addition, we did not include patients who received EBRT with definitive intention due to their rarity in our center. Moreover, the duration of follow-up was limited, and only the BFS was included. The current study did not assess other outcomes, such as OS and CSS, which require longer follow-ups to capture. Finally, adjuvant treatment approaches vary in different patients regarding radiation dose, ADT modality, drug dosage, and treatment duration, which will influence BCR evaluation but is challenging to balance. Despite all this, the results of our study reflect what is happening in the multimodal treatment era.

## Conclusion

We compared the efficacy of RP- and BT-based comprehensive treatment using two different BCR criteria. Our findings were different from previous studies, which revealed that the BFS of RP was comparable to that of BT with the Phoenix standard but superior to the surgical definition. Further analysis after PSM did not alter the comparative results. Well-designed prospective studies with clear BCR definitions are still needed to provide high-level evidence to facilitate clinical patient counseling and decision-making.

## Data Availability

The datasets used and analyzed during the current study are available from the corresponding author upon reasonable request.

## Abbreviations

RP: Radical prostatectomy
BT: Brachytherapy
LPCa: Localized prostate cancer
BCR: Biochemical recurrence
PSA: Prostate-specific antigen
PSM: Propensity score matching
BFS: Biochemical recurrence-free survival
ADT: Androgen deprivation therapy
EBRT: External beam radiotherapy
Neo-ADT: Neoadjuvant androgen deprivation therapy
RT: Radiotherapy
CSS: Cancer-specific survival
AUA: American Urologic Association
ASTRO: American Society for Therapeutic Radiology and Oncology
tPSA: Total prostate-specific antigen
BFS: biochemical recurrence-free survival
GS: Gleason score
cT-stage: clinical T stage
PPB: percentage of positive biopsy
TPS: treatment planning system
OS: Overall survival
PS: Propensity score
HR: Hazard ratio
CI: Confidence interval
